# Effectiveness of Integrated Emotional-Self Enhancement (IESE) program among staff nurses: protocol for a quasi-experimental study

**DOI:** 10.12688/f1000research.110656.1

**Published:** 2022-06-21

**Authors:** Monalisa Saikia, Linu Sara George, Bhaskaran Unnikrishnan, Anice George, N Ravishankar

**Affiliations:** 1 Department of Fundamentals of Nursing, Manipal College of Nursing, Manipal Academy of Higher Education, Manipal, Karnataka, 576104, India; 2 Kasturba Medical College, Mangalore, Manipal Academy of Higher Education, Manipal, Karnataka, 575001, India; 3 Department of Child Health Nursing, Manipal College of Nursing, Manipal Academy of Higher Education, Manipal, Karnataka, 576104, India; 4 Department of Bio-statistics, Vallabhbhai Patel Chest Institute, University of Delhi, New Delhi, Delhi, 110007, India

**Keywords:** emotional intelligence, staff nurses, nurses, nursing, emotional labor, intrinsic motivation, study protocol

## Abstract

**Background:** Staff nurses face frequent emotional situations in their work environment. The constant contact with suffering patients, and the busy work environment, pose tremendous stress on nurses’ physical and emotional health. The Emotional Intelligence skills of empathy, self-awareness, motivation, self-control, and keeping relationships, can help handle difficult emotions and allow nurses to work in an organized, calm, and professional way.

This study aims to implement and assess the effectiveness of a training program developed by the investigator, tailored to the mental and emotional needs of staff nurses who are working in an organization. The study also aims to observe any significant change, correlation, and association in the staff nurses’ level of emotional intelligence, intrinsic motivation, self-compassion, emotional labor, and nurse-in-charges’ and patients’ perception of nursing care after the program.

**Methods:** A quasi-experimental (one-group) study design was used in this study. The study will involve 80 staff nurses working in a selected hospital in India. The staff nurses will be selected from the hospital’s general wards using convenience sampling. For the current study, a quasi-experimental design will be used. The investigator will deliver a training program, divided into four sessions of two hours each. Data will be collected from the participants at baseline and 3-months pre-intervention; and post-test data will be collected immediately after the intervention, at 3-month, and 6-month follow-up, to observe any significant change in the study variables before and after the intervention.

**Results:** The current study primarily focuses on the vital aspect of developing emotional needs, for promoting a better work-life balance. Research findings from the study will significantly contribute to the evidence based Emotional Intelligence programs for staff nurses, and if proven effective, could be delivered extensively in the hospitals.

**Trial registration:** The study is registered in June 2019 under the Central Trial Registry of India ( CTRI/2019/08/020592).

## 1. Introduction

Nurses are faced with frequent emotional situations in their work environment. The constant contact with patients who are suffering, an emphasis on safety and quality of care, and the ever-engaging work environment, pose tremendous stress on the nurses’ health, physically and emotionally. Because of the many situational stresses faced by the nurses at work, their decision-making, concentration, and recall can be hindered, which may lead to an increase in error. The skills of an emotionally intelligent individual helps them to handle difficult emotions, especially in times of crisis, and allows them to act appropriately.

### 1.1 Background

Emotion is a complex feeling that results in physical and psychological changes which influence thoughts and behaviours ( Drigas et al., 2018). Emotions motivate our decisions, help store our memories, influence how we respond to a situation through our actions ( Keltner & Kring, 1998), and guide our interpersonal relationships ( Susskind et al., 2008).

Emotions are very fundamental to nursing practice. When nurses are aware of their own emotions, it allows them to perform optimally without controlling their behaviour. Research findings also show that emotions greatly influence professional relationships and impact healthcare workers’ decisions for their patients ( Freshwater et al., 2004).

The healthcare environment is always emotionally charged, where the nurses are faced with frequent emotional situations ( Reeves, 2005). As organizational and consumer demands are changing, nurses’ physical and emotional work is also increasing ( Tomar, 2016). The constant contact with patients, the imperative emphasis on safety and quality of care to the patients, and the work environment pose tremendous stress on nurses. Even in these stressful situations, nurses are expected to perform without emotional fluctuations or outbursts and also have the ability to know others’ and own emotions. This ascertains that the nurses who can manage their own emotions and understand others’ points of view can cope with stress much better than those who do not. But, controlling the emotions requires many psychological resources, which ultimately wears down the body and mind, and may lead to burnout ( Cheng et al., 2009), that can ultimately result in emotional distress, emotional labour, feelings of failure, demotivation, decreased quality of care, and conflicts with patients and colleagues ( Tomar, 2016).

Emotional Intelligence (EI) is the ability to identify, comprehend, and manage emotions in self and others, enhancing communication, solving problems, and managing conflicts ( Drigas et al., 2018). Although it is thought that health care providers already possess the skills necessary for being emotionally intelligent, research findings show that this is not always true ( Cheng et al., 2009). Srinivasan and Jebaseelan (2014), in their study, have found a low level of EI among nurses and recommended training to enhance EI among staff nurses, which would help them cope effectively with stress.

A Canadian integrative literature search covering 1995-2007 was conducted to ascertain the state of understanding about EI in nursing practice. The study found that many researchers believe that the nursing profession’s very essence requires nurses to be more emotionally intelligent and being aware of emotions is an essential nursing skill. The study concluded that EI skills and competencies could educate nurses about emotions and improve nursing care and human relationships, affecting patient outcomes ( Smith et al., 2009). The EI skills of empathy, self-awareness, motivation, self-control and maintaining relationships help handle emotions. Especially in times of crisis, the ability to self-regulate emotions positively allows nurses to work in an orderly, calm, and professional manner ( Chun et al., 2016). Evidence shows that EI has a significant correlation between self-compassion and the patients’ perception of nursing care ( Codier et al., 2013). Also, EI helps nurses in developing therapeutic relationships ( Cadman et al., 2001), influences patients’ care, their families, and the nurses’ overall well-being ( Raghubir, 2018).

An integrative systematic review explored the relationship between healthcare professionals’ EI and their caring behaviour towards patients. Studies published between the year 1995 to 2017 were searched and reviewed from digital databases such as PsycInfo, Medline, CINAHL, SSCI, SCI, and Scopus. A total of 22 studies which explored how healthcare workers’ EI impact patient’s caregiving was selected for review. The review results indicated that nurses’ EI has an impact on their physical and emotional caring behaviours for patients. The study recommended that interventions to enhance EI skills should be developed which may benefit the nurses as well as the patients ( Nightingale et al., 2018).

A simple PubMed search on the term “emotional intelligence” AND “nurs*” shows many published studies on EI. Most of the studies are cross-sectional which were done among nurse leaders and nursing students. The investigator found no recent study on EI training among staff nurses being published. Thus, the investigator has proposed the current study keeping in mind the knowledge gaps found after doing an extensive literature search, which are: i) limited studies have explored staff nurses’ EI and its relationship with their intrinsic motivation, emotional labour, and self-compassion; ii) very few studies exist on developing and assessing the effectiveness of a structured program aimed at enhancing EI, intrinsic motivation, self-compassion and reducing emotional labour for staff nurses.

The current study primarily focuses on the important aspect of developing emotional needs, such as emotional intelligence, intrinsic-motivation and self-compassion among staff nurses, for promoting a better work-life balance. Research findings from the current study will be a major contribution to the evidence-base for EI programs among staff nurses. If proven effective, it could be delivered extensively to hospitals.

### 1.2 AIM

This study’s primary goal is to develop, validate, and implement a structured Integrated Emotional Self Enhancement (IESE) Program among staff nurses. The program is tailored by keeping in mind the various mental and emotional stresses the staff nurses might experience in their everyday working environment. The study aims to examine the program’s impact on the staff nurses’ level of EI, Intrinsic Motivation (IM), Self-Compassion (SC), Emotional Labor (EL), and the patients’ and nurse-in-charges’ perception of nursing care before and after the program, using standardized and validated questionnaires.

## 2. Methods

### 2.1 Trial registration

The study is registered in June 2019 under the Central Trial Registry of India ( CTRI/2019/08/020592).

### 2.2 Design

The current study has a quasi-experimental (one-group) design, which involves staff nurses working in a selected hospital. The present study’s design is well suited, keeping in mind the work shifts of staff nurses and the number of staff nurses in the selected hospital. The current study has based its concept on three models: a) King’s conceptual system and theory of goal attainment ( Shanta & Connolly, 2013), b) GENOS EI model ( Palmer et al., 2009), c) motivation to care model ( Moody & Pesut, 2006).

### 2.3 Study site

The study will be conducted in a selected tertiary care hospital in Mangalore, India. This multispecialty hospital has outpatient and inpatient facilities that cater to patient care, teaching, and scientific research. The hospital has approximately a total of 450 staff nurses. The staff nurses have three shift duty hours viz. morning, evening, and night shift. To maintain the confidentiality of the study participants, the hospital name is not disclosed.

### 2.4 Study participants

The staff nurses working in the selected hospital’s general wards, who match the inclusion and exclusion criteria, will be considered as a potential study participant. All the nurses who provide their consent to participate will be considered for recruitment in the study. However, only those who fulfill the inclusion criteria will be included for the final sample. The IESE program will be delivered to all the recruited staff nurses. Also, data about the perception of nursing care will be collected from the patients who are under the direct care of the recruited nurse participants, and from the nurse-in-charges who are supervising the recruited nurses. The program will not be provided to the patients and nurse-in-charges. Table 1 shows the summary of the inclusion and exclusion criteria of study participants.

**Table 1.** Summary of inclusion and exclusion criteria.

	Nurse	Nurse-in-charge	Patient
**Inclusion criteria**	Staff nurses working in a selected hospital at Mangalore, Karnataka, India.	Nurse-in-charge working in a selected hospital at Mangalore, Karnataka, India.	Patients who are willing to participate.
	Staff nurses who are willing to participate in the study.	Nurse-in-charge who are willing to participate in the study.	Patients in the age group of 18-70 years.
			Patients who can read Kannada and English.
			Patients who are conscious.
**Exclusion criteria**	Staff nurses with earlier exposure to similar programs.	Nurse-in-charge working in psychiatric units, Out-patient-department, and Intensive Care Units.	Patients with current diagnosis of mental illness.
			Patients who are critically ill.
	Staff nurses working in psychiatric units, Intensive Care Units, and Out-patient Departments.		Patients who are admitted for less than 3 days.

### 2.5 Sample size and selection

For the current study, all staff nurses working in the general wards will have the probability of being selected. At a 10% dropout rate, 80% power, SD of 12.3 (taken after feasibility study), and clinically significant difference of 5, it was calculated that approximately 80 staff nurses should be recruited for the current study.

$$n=\frac{{\left[z_{1-\frac{\alpha }{2}}+z_{1-\beta }\right]}^{2}\sigma^{2}}{d^{2}}$$

$$∴n=\frac{{\left(1\cdot 96+0.84\right)}^{2}\times 12.3^{2}}{4^{2}}=75$$

$$\mathrm{At}\;\text{dropout rate of}\;10\%,n=\frac{75}{1-0.1}=83$$

### 2.6 Recruitment

For recruiting the participants, first, informed written consent to participate in the study will be collected from the staff nurses working in the selected hospital’s general wards. Then, to choose a sample of the consented staff nurses from each general ward, the sample will be matched against the inclusion and exclusion criteria. The nurses who do not fulfill the inclusion criteria will be excluded from the group.

Nurse-in-charges from each general ward will be taken conveniently. A written informed consent for participation will be taken before recruiting. The nurse-in-charges who are supervising the participating staff nurses at the time of data collection will only be considered for recruitment. If there is a ward that no staff nurses are taking part, the researcher will exclude the nurse-in-charge and patients from that unit.

Patients will also be selected conveniently. Only the patients who are receiving care from the staff nurses participating in the study, during the time of data collection, will be selected from each general ward. As the study is longitudinal, to collect data from the same patient in the in-patient department is very unlikely. Therefore, data from 80 patients will be collected at each (five) contact points, i.e., at baseline, 3-months pre-intervention, and immediately after the intervention, at 3-month, and 6-month follow-up.

### 2.7 Study intervention

The IESE Program was developed by the investigator with the help and collaboration of experts from the field of EI, Human Resource Development, Nursing Management, and Psychology. The investigator has also taken certificate training course on Emotional Intelligence from EquiPoise, Maharashtra. The experts in the above-mentioned field validated the program. The IESE program components are divided into four sessions:

Session 1: understanding emotions, emotional intelligence; importance of emotional intelligence.

Session 2: neuroscience behind emotional intelligence; emotional intelligence skills.

Session 3: emotional intelligence skills; management of difficult emotions.

Session 4: interpersonal relationships; motivation; empathy.

The program does not instruct the nurses on how to feel; rather, it encourages the nurses to reflect on their mental and emotional health. The investigator will deliver the program through lectures, Power Point presentations, group activities, situation analysis, videos, worksheets, and workbooks. A booklet will be given to the participants for future reference. These materials will be applied for a copyright by the authors. Hence, they have not been uploaded to an approved repository yet.

The investigator will divide the consented sample of staff nurses into four groups (20 nurses in each group) and will provide the training program to each group for four consecutive days, for two hours each day. If a participant misses any session, he/she will be accommodated in another group to complete the missed session. The sessions will be made flexible and accommodating.The investigator will make sure that the IESE program does not overlap with the staff nurses’ daily work schedule and hamper the hospital activities or disturb the patients. The nurse-in-charges, staff nurses, and the nurse-supervisor will be informed many weeks prior to starting the program to effectively make the duty roster. The investigator will collect e-mail address and phone numbers of the participants, for sending reminders about the sessions, and follow-up. Table 2 shows an overview of the time plan.

**Table 2.** Table showing the timeline of the current study.

	Study period							
	Enrolment	Pre-test		Intervention	Post-test			Analysis
Time point		T _0_ (Baseline)	T _-1_ (3-month)		T _1_ (Immediately after the intervention)	T _2_ (3-month)	T _3_ (6-month)	
Enrolment	✓							
Eligibility screen	✓							
Informed consent	✓							
Intervention				4 sessions of 2 hours each.				
Assessments of study variables		✓	✓		✓	✓		
Final data analysis and dissemination								

### 2.8 Study outcomes

Table 3 shows the primary and secondary outcomes, including the measurement. The secondary objectives/outcomes will be measured using the validated questionnaires at five time-points.

**Table 3.** Summary of the study outcomes and measures.

Outcomes	Description	Measure
Primary	To develop, validate and implement the structured Integrated Emotional-Self Enhancement Program	The researcher has developed a structured program keeping in mind the work structure and stress the nurses might feel in the work and personal environment. The developed program has been validated by the experts who are specialized from the field of nursing management, psychologists, psychiatrists, emotional intelligence, and human resources.
Secondary 1	To determine the impact of Integrated Emotional-Self Enhancement Program on staff nurses’ emotional intelligence	GENOS Emotional Intelligence Inventory (Concise version) ( Gignac, 2008).
Secondary 2	To determine the impact of Integrated Emotional- Self Enhancement Program on staff nurses’ emotional labor	Emotional Labor Scale ( Brotheridge & Lee, 2002).
Secondary 3	To determine the impact of Integrated Emotional-Self Enhancement Program on staff nurses’ self-compassion	Self-compassion Scale ( Neff, 2010).
Secondary 4	To determine the impact of Integrated Emotional-Self Enhancement Program on staff nurses’ intrinsic motivation	Intrinsic Motivation Assessment Scale (developed by the investigator)
Secondary 5	To examine any change in the patients’ perception of nursing care	Patients’ Perception of Nursing Care Scale (developed by the investigator)
Secondary 6	To examine any change in nurse-in-charges' perception of nursing care	Nurse-in-charges’ Perception of Nursing Care Scale (developed by the investigator)

### 2.9 Data collection

Data from the staff nurses, patients, and nurse-in-charges will be collected at baseline and 3-month pre-intervention, and immediately after the intervention, at 3-month, and 6-month follow-up by the investigator. Secondary outcome data will be collected via pen-and-paper format. The questionnaires will be completed by the participants on the same day. The investigator will always be present with the participants when they fill in the questionnaire and will provide help to understand the questionnaire’s statements if needed. All the participants will be given a unique code to maintain confidentiality and to make sure that only the participants who have consented to participate complete the questionnaires. The investigator will collect the questionnaires on completion and store them safely in a folder. The investigator has considered an attrition rate of 10% while calculating the sample size, to account for participants who discontinue the intervention or follow-ups. Also, the investigator will maintain an attendance sheet of the participants. Reminders will be sent, and calls will be made to the study participants about upcoming and missed sessions.

### 2.10 Data collection measures/instruments

The tools will be administered to participants at each time point. The investigator has taken permission from the respective authors for using the standardized tools. All the tools/instruments were chosen for their usefulness and appropriateness to fulfil the current study’s objectives. All the standardized instruments have been previously used in health and occupational research. The tools developed by the investigator have been validated by subject experts. Also, feasibility and pilot tests were done to measure the reliability of all the instruments which are to be used in the study.

Demographic variables to be collected are age, gender, education qualification, marital status, number of family members, personal annual income, type of job (full time/part-time), and ward/unit.

GENOS EI Inventory (concise version) will be used to assess the staff nurses’ EI. The tool focuses upon the measurement of the frequency of typicality with which an individual exhibits EI, and has 31 items across seven dimensions of emotional awareness of self and others, emotional management of self and others, emotional expression, and emotional self-control. The items are scored on a five-point Likert scale, from ‘almost never’ to ‘almost always’. The test-re-test reliability score of the tool is 0.9 ( Palmer et al., 2009; Gignac, 2008, 2010).

The Emotional Labour Scale consists of 15 items with the subscales like duration (1 item), frequency (3 items), intensity (2 items), variety (3 items), surface acting (3 items) and deep acting (3 items). The reliability of the tool is 0.83. Respondents are asked to rate “on an average day at work how frequently” they performed interpersonal behaviours on a 5-point Likert-type response scale (1 = never, 5 = always). The higher the score, the higher the emotional labour ( Brotheridge & Lee, 2002).

The Self Compassion Scale developed by Kristen Neff ( Neff, 2010) will be used in the current study. The test-re-test reliability score of the scale is 0.92. The scale has 12 items that are scored on a five-point Likert scale from “almost never” to “almost always”. The subscales measure self-kindness, self-judgement, humanity, isolation, and mindfulness. Average self-compassion score of 1-2.5 indicates low self-compassion, 2.5-3.5 indicates moderate, and 3.5-5.0 indicates a high level of self-compassion.

Intrinsic motivation will be measured using the Intrinsic Motivation Scale (IMS) which is developed by the investigator. The scale is developed based on Self Determination Theory ( Ryan & Deci, 2000), Mc Clelland Motivation Theory ( Pardee, 1990), and Motivation to Care Model ( Moody & Pesut, 2006). The scale’s major components are autonomy, competence, relatedness, knowledge, skills, and inner drive. There are 28 items in the IMS, which has been validated by experts with a Content Validity Index of 0.87 and a Cronbach’s alpha reliability score of 0.91.

The Patients’ Perception tool is developed by the investigator to assess the patients’ perception of nursing care before and after the intervention program. The tool has ten items that are scored on a Likert scale from “strongly agree” to “strongly disagree”. The tool has been validated by experts and translated into Kannada (Local language). The Cronbach’s alpha reliability score and Content Validity Index of the tool is 0.81 and 0.87 respectively.

The investigator developed the nurse-in-charges’ perception of nursing care tool to assess the nurse supervisors’ perception towards nursing care before and after the intervention program. The tool has ten items that are scored on a Likert scale from “strongly agree” to “strongly disagree”. The tool has been validated by experts. The Cronbach’s alpha reliability score and Content Validity Index of the tool is 0.93 and 0.87 respectively.

### 2.11 Data management

All the questionnaires are paper-and-pen based. So, the data obtained will be entered manually into Microsoft Excel (version 16.0) sheets and SPSS (version 16) and will be stored under a folder with a password. The investigator will rectify the mistake if a participant signs their name or any other identifying information on the questionnaire at the time of data collection. The data will all be stored safely on a secure server for further analysis and reference, and the paper copies will be stored safely for five years after the study’s completion.

### 2.12 Statistical analysis

Mean and standard deviation will be used to describe measurement data. Frequencies and percentages will be used to represent count data. Repeated measures ANOVA will be used to analyze the effect of the training program. Chi-square will be used to see if an association between the variables before is after the training program.

### 2.13 Risk monitoring and referral

The current study is safe, considering there are no invasive procedures for the intervention. There are no direct risks associated with taking part in the current study. However, participants may feel uncomfortable or stressed while attending the program or while answering the questionnaires, of which the investigator will be very observant about. Also, in the current pandemic state, the investigator will be very vigilant and cautious of all the norms and regulations to be followed to eliminate the risk of participants getting COVID-19 infection. In any crisis, the participant will be flagged and at once referred for counselling or any other support as needed. During the study, the participants (staff nurses) are needed to attend sessions on IESE program and provide responses in the questionnaires given to them. Before recruiting the study participants, the investigator will provide them with the study’s details, including the purpose, details of the program, potential risks, and benefits of being a participant. Similarly, the patients and nurse-in-charges will also be given information in detail about the program and their role in the study.

## 3. Ethics and consent

The investigator has received approval from the university ethics committee (Kasturba Medical College and Kasturba Hospital Institutional Ethics Committee, approval number 427/2019; Institutional Ethics Committee Kasturba Medical College, Mangalore, approval number- IEC KMC MLR 08-19/331) in 2019 and has received written permission from the selected hospital authorities to conduct the study. The investigator will provide all the staff nurses, patients, and nurse-in-charges with a participant information sheet and a written consent form will be collected from the study participants. The participant information sheet will hold all the details of the study, including the aims, objectives, details about the intervention or absence of it, and the risks and benefits of participating. No one will be forced or compelled to participate in the study. The participants can withdraw from the study at any time during the study without penalty.

## 4. Dissemination

The investigator intends to disseminate the study results by publishing in peer-reviewed Scopus indexed journals and present papers at scientific conferences.

## 5. Confidentiality and access to data

The study data will be safely stored in a digital format on a secure server for five years after completion of the study. The raw data will be accessible to only the research team members and will be kept in a password-protected file. However, de-identified data can be made accessible on request.

## 6. Discussion

Nurses work in an environment that is always emotionally charged. They work with patients and their families who are most often dealing with demanding situations, emotionally and physically. Being self-aware of one’s own emotions, empathizing, having meaningful relationships, and being compassionate are important aspects of professional nursing. Understanding EI is incredibly significant to the nursing profession because of its nature and the complexities and challenges it brings with it. Many researchers have recommended conducting studies by developing and providing a structured program to enhance nurses’ EI. The investigator believes that the intervention program in the study would help enhance the nurses’ EI, which would influence their intrinsic motivation and self-compassion, thereby reducing their emotional labor, which would eventually positively impact their professional and personal life, as well as in the patient outcome.

## 7. Limitations

One of the major limitations of the current study is the study design; the study is quasi-experimental with only one group and no control arm. The study could not select a more robust design like a randomized control trial because of the lack of similar hospitals in terms of infrastructure, workforce, and working schedule. Compliance to the training program could also be a huge limitation to the study. However, measures will be taken to address this limitation. The investigator will reinforce the participants to comply with the training program and will encourage them to apply the learnings in their daily life by sending out regular emails and messages. Another limitation is the small sample size of 80 staff nurses and the chances of participant attrition during subsequent follow-up. To tackle this limitation, an attrition of 10% is estimated while calculating sample size. The investigator will also send reminder e-mails and messages to participants before every follow-up. All limitations can veil the true effect of the program. However, considering the current state of COVID-19 pandemic, where staff nurses and healthcare professionals are scarce in general, and to avoid large gatherings that can put the participants’ health at risk, the investigator believes that the current study design and sample size will suffice the study objectives.

## 8. Conclusion

The IESE program can have a significant impact on staff nurses’ personal and professional lives, which can have a positive effect on the patient care outcome. Such programs on EI are a need of the hour, as healthcare workers and nurses are facing great stress in the face of COVID-19 pandemic. The findings of the current study will add to the research and development of emotional intelligence training programs among nursing and healthcare professionals.

## 9. Study status

The study intervention has been delivered to the study participants. The follow-up data collection is expected to complete in April - May 2022.

## Author contributions

Saikia M conceived of the study; George LS, George A & Unnikrishnan B initiated the study design; N Ravishankar provided expertise in statistics and study design. All authors contributed to refinement of the study protocol and approved the final manuscript.

## Roles and responsibilities of committees

i. ***Principal investigator and co-authors***:
  • Design and conduct of the study.
  • Preparation of protocol and revisions
  • Preparation of intervention modules and materials
  • Publication of study reports
ii. ***Institutional Ethics Committee***:
  • Monitoring study
  • Audit of 6 monthly feedback from the principal investigator
  • Data verification

## Protocol amendments

Any modifications to the protocol which may impact on the conduct of the study will be informed and amended by the Institutional Ethics Committee of the current study setting.

## Data availability

### Underlying data

No underlying data are associated with this protocol.

### Extended data

figshare: Effectiveness of Integrated Emotional Self Enhancement (IESE) Program among staff nurses: protocol for a quasi-experimental study. https://doi.org/10.6084/m9.figshare.19428638 ( Saikia *et al*., 2022).

The project contains the following extended data:

• English PIS (nurses).doc (Participant Information Sheet for nurses)
• English PIS (nurse-in-charge).doc (participant information sheet for nurse-in-charge)
• English PIS (patients).doc (Participant information sheet for patients)
• intrinsic motivation.docx (Intrinsic motivation scale, developed by the investigator)
• nurse incharge’s perception.docx (nurse-in-charges’ perception of nursing care scale, developed by the investigator)
• patients perception.docx (patients’ perception of nursing care scale, developed by the investigator)
• demographic proforma (patients).docx (demographic proforma for patients)
• demographic proforma (Staff nurses).docx (demographic proforma for staff nurses)
• demographic proforma (nurse-in-charge).docx (demographic proforma for nurse-in-charges)

Data are available under the terms of the Creative Commons Attribution 4.0 International license (CC-BY 4.0).

Copyright will be applied for the booklet, workbook, and teaching material which will be developed by the investigators, and hence these materials have not been uploaded to the repository yet.

## Reporting guidelines

figshare: SPIRIT checklist for ‘Effectiveness of Integrated Emotional-Self Enhancement (IESE) Program among staff nurses: Protocol for a quasi-experimental study’. https://doi.org/10.6084/m9.figshare.19429757
