# Effectiveness of Integrated Emotional-Self Enhancement (IESE) program among staff nurses: protocol for a quasi-experimental study

**DOI:** 10.12688/f1000research.110656.3

**Published:** 2023-10-06

**Authors:** Monalisa Saikia, Linu Sara George, Bhaskaran Unnikrishnan, Anice George, N Ravishankar

**Affiliations:** 1 Department of Fundamentals of Nursing, Manipal College of Nursing, Manipal Academy of Higher Education, Manipal, Karnataka, 576104, India; 2 Kasturba Medical College, Mangalore, Manipal Academy of Higher Education, Manipal, Karnataka, 575001, India; 3 Department of Child Health Nursing, Manipal College of Nursing, Manipal Academy of Higher Education, Manipal, Karnataka, 576104, India; 4 Department of Bio-statistics, Vallabhbhai Patel Chest Institute, University of Delhi, New Delhi, Delhi, 110007, India

**Keywords:** emotional intelligence, staff nurses, nurses, nursing, emotional labor, intrinsic motivation, study protocol

## Abstract

**Background:** Staff nurses face frequent emotional situations in their work environment. The constant contact with suffering patients, and the busy work environment, pose tremendous stress on nurses’ physical and emotional health. The Emotional Intelligence skills of empathy, self-awareness, motivation, self-control, and keeping relationships, can help handle difficult emotions and allow nurses to work in an organized, calm, and professional way.

This study aims to implement and assess the effectiveness of a training program developed by the investigator, tailored to the mental and emotional needs of staff nurses who are working in an organization. The study also aims to observe any significant change, correlation, and association in the staff nurses’ level of emotional intelligence, intrinsic motivation, self-compassion, emotional labor, and nurse-in-charges’ and patients’ perception of nursing care after the program.

**Methods:** A quasi-experimental (one-group) study design was used in this study. The study will involve 80 staff nurses working in a selected hospital in India. The staff nurses will be selected from the hospital’s general wards using convenience sampling. For the current study, a quasi-experimental design will be used. The investigator will deliver a training program, divided into four sessions of two hours each. Data will be collected from the participants at baseline and 3-months pre-intervention; and post-test data will be collected immediately after the intervention, at 3-month, and 6-month follow-up, to observe any significant change in the study variables before and after the intervention.

**Results:** The current study primarily focuses on the vital aspect of developing emotional needs, for promoting a better work-life balance. Research findings from the study will significantly contribute to the evidence based Emotional Intelligence programs for staff nurses, and if proven effective, could be delivered extensively in the hospitals.

**Trial registration:** The study is registered in June 2019 under the Central Trial Registry of India ( CTRI/2019/08/020592).

## 1. Introduction

Nurses are faced with frequent emotional situations in their work environment which may hinder their decision-making, concentration, and recall, and may lead to an increase in errors. Thus, the skills of emotion regulation may help them in handling difficult emotions, especially in the times of crisis, and allow them to act appropriately.

### 1.1 Background

Emotion is a complex feeling that results in physical and psychological changes which influence thoughts and behaviours ( Drigas & Papoutsi, 2018), motivate our decisions ( Keltner & Kring, 1998), and guide our interpersonal relationships ( Susskind, Lee, Cusi, Feiman, Grabski, & Anderson, 2008). Emotions are fundamental to nursing practice and play a vital role in the interaction with colleagues and patients. It is through the exchange and perception of emotions that nurses and patients initiate, establish, and maintain a therapeutic relationship. Therefore, it is essential that nurses understand and manage their own emotions and are skilled in managing others’ emotions.

Nurses face various difficult events and emotions due to the nature of the job itself, which puts them in a vulnerable position ( Lee, 2019). Additionally, nurses are expected to control their emotional reactions while calming their patients’ distress, show empathy, be always amiable ( Ryan & Deci, 2000), express positive emotions, and suppress negative emotions ( Glomb & Tews, 2004). Such experiences can be overwhelming, emotionally laborious, and expose nurses to “psychological strain” ( Zaghini, Fiorini, Piredda, Fida, & Sili, 2020). When nurses experience such tremendous occupational stress, the motivation to engage in work and serve others lowers, potentially leading to a loss in productivity ( Jiang, Jia, Zhang, Li, Song, & Yu, 2021) and ultimately negatively affecting their quality of care ( Varghese, George, Kondaguli, Naser, Khakha, & Chatterji, 2021).

In recent years, various emotional well-being components/concepts have garnered the attention of scientists and have been proven to impact a variety of outcomes in healthcare workers such as optimal physical and psychological health, intelligent decision-making, effectively managing workload, burnout and stress ( Hotchkiss, 2018), as well as improved ability to care for patients with greater compassion and more sustainably, sensitivity, and empathy and improved interpersonal skills ( Søvold, Naslund, Kousoulis, Saxena, Qorondleh, Grobler, & Münter, 2021). One such concept is Emotional Intelligence (EI) which can be defined as an individual’s ability to understand others and their own emotions to make intelligent decisions. Previous studies have suggested that individuals with high EI have a set of emotional aptitudes and competencies that enable them to evaluate and regulate their as well as other’s emotions (e.g., anger) to reach a variety of adaptive outcomes or emotional states (e.g., motivation) ( O’Connor, Hill, Kaya & Martin, 2019). The available literature in Nursing also recognizes EI as a crucial element in providing patient-centred care ( Giménez-Espert & Prado-Gascó, 2018), and suggests that nurses with higher EI experiences less professional stress, perform better at work, have better communication skills, are empathetic and solves problems more effectively ( Kahraman & Hicdurmaz, 2016). These findings emphasize the importance of EI in nursing.

Intrinsic motivation (IM), self-compassion (SC) and emotional labour (EL) have also been closely linked with EI in the available literature. Nurses with higher emotional regulation skills were found to have high IM ( Lelorain, Bachelet, Goncalves, Wortel, Billes, Seillier, Bertin, & Bourgoin, 2018). This suggests that interventions aiming at improving nurses’ emotional regulation/EI skills may also improve their IM.

Kristen Neff (2003) defined SC as being open to and moved by one’s suffering, experiencing feelings of caring and kindness toward oneself, taking an understanding non-judgmental attitude toward one’s inadequacies and failures and recognizing that one’s own experience is part of the common human experience. Neff (2003) also suggests that individuals who are capable of self-compassion are kind to themselves and accept reality, and in doing so hold a balanced awareness of their thoughts and feelings by being emotionally intelligent. Within the nursing literature SC is found to be positively associated with higher EI ( Heffernan, Griffin, McNulty, & Fitzpatrick, 2010; Kousar, Kousar, Afzal, Waqasr, & Gilani, 2017; Joseph & Elias, 2019), higher IM ( Kotera, Taylor, Fido, Williams, & Tsuda-McCaie, 2021), greater well-being ( Durkin & Beaumont, 2016), lower compassion fatigue and burnout ( Beaumont, Irons, Rayner, & Dagnall, 2016; Dev, Fernando, Lim, Consedine, 2018; Durkin & Beaumont, 2016).

As mentioned earlier, nurses are always in direct contact with patients who are suffering. In this interaction, nurses are always expected to keep a check on their as well as patients’ emotions. Pam Smith defined this as emotional labour, which is the act or skills that are involved in caring for and recognizing the emotions of others and not only the emotions experienced by nurses ( Freitas, Costa, Diogo, & Gaiva, 2021). Available literature indicates that nurses experience mild to moderate EL, which if remains unchecked, leads to emotional dissonance, emotional exhaustion ( Badolamenti, Sili, Caruso, & Fida, 2017), stress and burnout ( Delgado, Upton, Ranse, Furness, & Foster, 2017). However, EL is also recognized as an essential characteristic of nurse-patient interaction ( Delgado, Upton, Ranse, Furness, & Foster, 2017), and the caring process ( Tuna & Baykal, 2017) which helps nurses in establishing and maintaining a trusting relationship with patients ( Yeh, Chen, Yuan, Chou, & Wan, 2020).

Despite the obvious emotional element of nurses’ work, there remains a dearth of professional acknowledgement and literature on emotional work in nursing practice ( Williams, 2013). After performing a thorough literature search, the investigator identified a few research gaps. First, very few studies in the available literature have implemented and tested interventions designed to enhance nurses’ emotional intelligence, intrinsic motivation, self-compassion, and emotional labour. Second, studies that have assessed the effectiveness of an intervention to improve nurses’ EI, IM, SC and EL for a longer follow-up are very minimal. Third, limited studies have explored the relationship between nurses’ EI, IM, SC, and EL. Fourth, although there is enough evidence indicating that nurses’ ability to regulate emotions influences the quality of care ( Khademi, Abdi, Saeidi, Piri, & Mohammadian, 2021), no previous studies have assessed the perception of nursing care after the nurses underwent emotional-wellbeing training. It was also observed that studies on patients’ perception of nursing care are inadequately explored in low-income countries ( Gishu, Weldersadik, & Tekleab, 2019; Kewi, Tasema, & Negussie, 2018; Dikmen & Yilmaz, 2017), as well as no studies were found that assessed nurse-in-charges’/supervisors’ perception of nursing care.

Recent studies suggest that more than 87.6% of nurses in India experience stress in their workplace ( Bai & Ravindran, 2020). Factors such as work overload, poor staffing, long shift hours, dealing with death and suffering, and rapidly changing technology, lead to increased fatigue, low motivation, increased error ( Wu, Zhu, Wang, Wang, & Lan, 2007), excessive anger and worry ( Kane, 2009), anxiety and depression ( Chaudhury, Mazumdar, & Mehta, 2018). In 2015, the United Nations (UN) Sustainable Development Goals (SDG) defined mental health as a priority for global development for the next 15 years. As the patient population is increasing, with ever-increasing stressors for nurses, it is a need of the moment that mental health and emotional wellness are paid due consideration and are strengthened ( WHO, 2022) by using both general and targeted evidenced-based interventions, and by creating and promoting strength-based intervention and preventative programs. Remarkably, studies that focus on improving positive emotions, motivation and behaviors of healthcare workers are rare ( vanDorssen-Boog, vanVuuren, DeJong, & Veld, 2021). As EI, IM, SC ( Fabio & Saklofske, 2021) and EL are among the many identified primary prevention resources, linked with psychological health and well-being in previous research, interventions aimed at supporting and strengthening nurses’ personal and psychological resources would not only aid in enhancing their emotional regulation, emotional well-being, and coping mechanisms, but they may also improve patient care. The investigator of this study has weaved these components of emotional well-being together with the purpose to strengthen them through a training program for promoting a better work-life balance.

### 1.2 AIM

This study’s primary aim was to develop, validate, and implement a structured Integrated Emotional-Self Enhancement (IESE) Program among staff nurses and examine the program’s impact on the staff nurses’ EI, IM, SC and EL, as well as on the patients’ and nurse-in-charges’ perception of nursing care.

## 2. Methods

### 2.1 Trial registration

The study is registered in June 2019 under the Central Trial Registry of India ( CTRI/2019/08/020592).

### 2.2 Design

The current study has a quasi-experimental (one-group) design, which involves staff nurses working in the general wards of a selected hospital. The present study’s design is well suited, keeping in mind the work shifts of staff nurses and the number of staff nurses in the selected hospital. To derive a better understanding of whether intervention on emotional well-being components would affect staff nurses and the perception of patients and nurse-in-charges, the current study has based its concept on Neuman’s System Model and King’s Interacting Systems Theory to design the conceptual framework. Both of these theories have been proven effective in providing guidance for applying the theoretical understanding to practical clinical nursing situations to improve practice ( Montano, 2021; Shanta & Connoly, 2013).

### 2.3 Study site

The study will be conducted in a selected tertiary care hospital in Mangalore, India. This multispecialty hospital has outpatient and inpatient facilities that cater to patient care, teaching, and scientific research. The hospital has approximately a total of 450 staff nurses. The staff nurses have three shift duty hours viz. morning, evening, and night shifts. To maintain the confidentiality of the study participants, the hospital name is not disclosed.

### 2.4 Study participants

The staff nurses working in the selected hospital’s general wards, who match the inclusion and exclusion criteria, will be considered as a potential study participant. All the nurses who provide their consent to participate will be considered for recruitment in the study. However, only those who fulfill the inclusion criteria will be included for the final sample. The IESE program will be delivered to all the recruited staff nurses. Also, data about the perception of nursing care will be collected from the patients who are under the direct care of the recruited nurse participants, and from the nurse-in-charges who are supervising the recruited nurses. The program will not be provided to the patients and nurse-in-charges. Table 1 shows the summary of the inclusion and exclusion criteria of study participants. The confounding factors will be controlled by applying an appropriate research design, communicating clearly with the participants and stakeholders, and by making the duty roster of staff nurses well in advance.

**Table 1.** Summary of inclusion and exclusion criteria.

	Nurse	Nurse-in-charge	Patient
**Inclusion criteria**	Staff nurses working in a selected hospital at Mangalore, Karnataka, India.	Nurse-in-charge working in a selected hospital at Mangalore, Karnataka, India.	Patients who are willing to participate.
	Staff nurses who are willing to participate in the study.	Nurse-in-charge who are willing to participate in the study.	Patients in the age group of 18-60-years.
			Patients who can read Kannada and English.
			Patients who are conscious.
**Exclusion criteria**	Staff nurses with earlier exposure to similar programs.	Nurse-in-charge working in psychiatric units, Out-patient-department, and Intensive Care Units.	Patients with current diagnosis of mental illness.
			Patients who are critically ill.
	Staff nurses working in psychiatric units, Intensive Care Units, and Out-patient Departments.		Patients who are admitted for less than 3 days.

### 2.5 Sample size and selection

For the current study, all staff nurses working in the general wards will have the probability of being selected. At a 20% dropout rate, 80% power, SD of 12.3 (taken after feasibility study), and clinically significant difference of 5, it was calculated that approximately 63 staff nurses should be recruited for the current study. However, to reduce the chances of higher attrition due to the unpredictable nature of the hospital environment, the researchers decided to recruite 80 staff nurses.

$$n=\frac{{\left[Z_{1-\frac{\alpha }{2}}+Z_{1-\beta }\;\right]}^{2}\sigma^{2}}{d^{2}}$$

*n* = minimum sample size required

$Z_{1-\frac{\alpha }{2}}$ = 1.96 at *α* = 0.05

$Z_{1-\beta }$ = 0.84 at 80% power

*σ* = population SD of the outcome variable = 12.3

*d* = clinically significant difference = 5

$$∴n=\frac{{\left(1.96+0.84\right)}^{2}{\left(12.3\right)}^{2}}{5^{2}}=50$$

With 20% drop out rate

$$n*=\frac{n}{1-0.3}\;=\frac{50}{0.2}=63\;\left(\text{approx}.\right)$$

As nurse-in-charges of all the general wards of the selected hospital will be approached for their consent to participate using complete enumeration, a sample size calculation was not performed. Similarly, a sample calculation was not performed for patients, because only those in-patients who will be receiving care from the recruited staff nurses will be approached for their consent to participate in the study.

### 2.6 Recruitment

For recruiting the participants, first, informed written consent to participate in the study will be collected from the staff nurses working in the selected hospital’s general wards. Then, to choose a sample of the consented staff nurses from each general ward, the sample will be matched against the inclusion and exclusion criteria. The nurses who do not fulfil the inclusion criteria will be excluded from the study.

Nurse-in-charges from each general ward will be taken conveniently using complete enumeration. The nurse-in-charges who are supervising the participating staff nurses at the time of data collection will only be considered for recruitment. If there is a ward from where no staff nurses are taking part, the researcher will exclude the nurse-in-charge and patients from that ward. A written informed consent for participation will be taken before recruiting.

Patients will also be selected conveniently. Only the patients who are receiving care from the staff nurses participating in the study, during the time of data collection, will be selected from each general ward. One patient (who provides their consent) for each nurse will be selected for the study (1:1). As the study is longitudinal, collecting data from the same patient in the in-patient department is very unlikely. Therefore, data from 80 patients will be collected at each (five) contact point, i.e., at baseline, 3-months pre-intervention, and immediately after the intervention, at 3-month, and 6-month follow-up.

### 2.7 Study intervention

The IESE Program was developed by the investigator with the help and collaboration of experts from the field of EI, Human Resource Development, Nursing, and Psychology. The investigators’ personal experience working as a mental health nurse and a thorough literature search served as the foundation for need analysis and base for intervention development. Also, before developing the training program, the investigator interacted with a group of nurses from the study setting as well as other hospitals to do a need assessment. The investigator asked them about the factors that induces stress, their emotional experiences and emotional requirements at work, and the ways they handle stressful situations. The investigator also interacted with patients and nurse-in-charges and nursing supervisors of two hospitals to understand what their expectations are from nursing services. This allowed the investigator to identify and understand practices and gaps in real world. By combining the findings from the need assessment, and critical observation the framework of the training program was developed. Drafts of the IESE program was shared among the above mentioned experts several times for their suggestions and modifications were made as suggested before finalizing the content of the program. The program was also pilot tested among 12 staff nurses in a different setting to establish its credibility and assess its effectiveness. The investigator has also taken a hands-on certificate training course on Emotional Intelligence to gain an understanding of Emotional Intelligence techniques under the supervision of experts in the field of Emotional Intelligence from EquiPoise, Maharashtra, which is a certified Emotional Intelligence training institute in India.

The IESE program does not instruct the nurses on how to feel; rather, it encourages the nurses to reflect on their mental and emotional health. The investigator will deliver the program through lectures, Power Point presentations, group activities, situation analysis, videos, worksheets, and workbooks. A booklet will be given to the participants for future reference. These materials will be applied for a copyright by the authors. Hence, they have not been uploaded to an approved repository yet.

The IESE program components are divided into four sessions. An outline of the sessions is provided in Table 2.

**Table 2.** Table showing outline of the IESE Program.

Session	Outline of the content	Duration
I	1. Understanding emotions, intelligence and emotional intelligence a. What is emotion? b. Views on emotion: the old and new paradigms c. How are emotions formed? d. The Plutchik model of emotion e. What is intelligence? f. The types of intelligence 2. The concept of Emotional Intelligence a. The importance of emotional intelligence	2 hour
II	1. The neuroscience behind emotional intelligence a. Basic brain structure and their function b. Parts of the brain that are associated with emotions c. Emotional/amygdala hijack 2. GENOS Model of Emotional Intelligence 3. Core components of emotional intelligence skills	2 hours
III	1. Emotional literacy 2. Interpersonal relationship, sympathy and empathy a. Importance of empathy in healthcare b. Effective interpersonal communication techniques 3. Concept of Emotional Labour and Self-Compassion	2 hours
IV	1. Concept of motivation and developing gratitude 2. Management of difficult emotions: anger and stress 3. Management of difficult emotions: anxiety and sadness	2 hours

To provide the IESE program without disrupting the work environment of the hospital, the consented nurses will be divided into smaller groups of 5-10, so that the participants engage in the activities of the training program with more efficiency. Each group will be given the same IESE program in similar setting and using similar techniques. Nurses in each group will complete a total of eight hours of training (two hours each day for four days) at the end of the intervention program. If a participant misses any session, he/she will be accommodated in another group to complete the missed session. The sessions will be made flexible and accommodating. The investigator will make sure that the IESE program does not overlap with the staff nurses’ daily work schedule and hamper the hospital activities or disturb the patients. The nurse-in-charges, staff nurses, and the nurse-supervisors will be informed many weeks prior to starting the program to effectively make the duty roster. The investigator will collect the e-mail address and phone numbers of the participants, for sending reminders about the sessions, and follow-up. Table 3 shows an overview of the time plan. The project will be implemented from approximately January 2021 to May 2022, with a total duration of 17 months (which includes data collection and intervention implementation).

**Table 3.** Table showing the timeline of the current study.

	Study period							
	Enrolment	Pre-test		Intervention	Post-test			Analysis
Time point		T _0_ (Baseline)	T _-1_ (3-month)		T _1_ (Immediately after the intervention)	T _2_ (3-month)	T _3_ (6-month)	
Enrolment	✓							
Eligibility screen	✓							
Informed consent	✓							
Intervention				4 sessions of 2 hours each.				
Assessments of study variables		✓	✓		✓	✓	✓	
Final data analysis and dissemination								

### 2.8 Study outcomes

Table 4 shows the primary and secondary outcomes, including the measurement. The secondary objectives/outcomes will be measured using the validated questionnaires at five time-points.

**Table 4.** Summary of the study outcomes and measures.

Outcomes	Description	Measure
Primary	To develop, validate and implement the structured Integrated Emotional-Self Enhancement Program	The researcher has developed a structured program keeping in mind the work structure and stress the nurses might feel in the work and personal environment. The developed program has been validated by the experts who are specialized from the field of nursing management, psychologists, psychiatrists, emotional intelligence, and human resources.
Secondary 1	To determine the impact of Integrated Emotional-Self Enhancement Program on staff nurses’ emotional intelligence	GENOS Emotional Intelligence Inventory (Concise version) ( Palmer, Stough, Harmer, & Gignac, 2009).
Secondary 2	To determine the impact of Integrated Emotional- Self Enhancement Program on staff nurses’ emotional labor	Emotional Labor Scale ( Brotheridge & Lee, 2002).
Secondary 3	To determine the impact of Integrated Emotional-Self Enhancement Program on staff nurses’ self-compassion	Self-compassion Scale ( Neff, 2003).
Secondary 4	To determine the impact of Integrated Emotional-Self Enhancement Program on staff nurses’ intrinsic motivation	Intrinsic Motivation Assessment Scale (developed by the investigator)
Secondary 5	To examine any change in the patients’ perception of nursing care	Perception of Nursing Care Scale (developed by the investigator)
Secondary 6	To examine any change in the nurse-in-charges’ perception of nursing care.	Perception of Nursing Care Scale (developed by the investigator)

### 2.9 Data collection

Data from the staff nurses, patients, and nurse-in-charges will be collected at baseline and 3-month pre-intervention, and immediately after the intervention, at 3-month, and 6-month follow-up by the investigator. Multiple baselines enable an assessment of whether changes occur after or during an intervention, rather than before or as a result of any extraneous factor ( Dundas, Binder, Hansen, & Stige, 2017). The questionnaires will be completed by the participants on the same day. The investigator will always be present with the participants when they fill in the questionnaire and will provide help to understand the questionnaire’s statements if needed. All the participants will be given a unique code to maintain confidentiality and to make sure that only the participants who have consented to participate complete the questionnaires. The investigator will collect the questionnaires on completion and store them safely in a folder. The investigator has considered an attrition rate of 20% while calculating the sample size, to account for participants who discontinue the intervention or follow-ups. Also, the investigator will maintain an attendance sheet of the participants. Reminders will be sent, and calls will be made to the study participants about upcoming and missed sessions.

### 2.10. Data collection measures/instruments

The tools will be administered to participants at each time point. The investigator has taken permission from the respective authors for using the standardized tools. All the tools/instruments were chosen for their usefulness and appropriateness to fulfil the current study’s objectives. All the standardized instruments have been previously used in health and occupational research. The tools developed by the investigator have been validated by subject experts. Also, feasibility and pilot tests were done to measure the reliability of all the instruments which are to be used in the study.

Demographic variables to be collected are age, gender, education qualification, marital status, type of family, personal annual income, type of job (full time/part-time), and ward/unit.

GENOS EI Inventory (concise version) will be used to assess the staff nurses’ EI. The tool focuses upon the measurement of the frequency of typicality with which an individual exhibits EI, and has 31 items across seven dimensions of emotional awareness of self and others, emotional management of self and others, emotional expression, and emotional self-control. The items are scored on a five-point Likert scale, from ‘almost never’ to ‘almost always’. The test-re-test reliability score of the tool is 0.9 ( Palmer, Stough, Harmer, & Gignac, 2009).

The Emotional Labour Scale consists of 15 items with the subscales like duration (1 item), frequency (3 items), intensity (2 items), variety (3 items), surface acting (3 items) and deep acting (3 items). The reliability of the tool is 0.83. Respondents are asked to rate “on an average day at work how frequently” they performed interpersonal behaviours on a 5-point Likert-type response scale (1 = never, 5 = always). The higher the score, the higher the emotional labour ( Brotheridge & Lee, 2002).

The Self Compassion Scale developed by Kristen Neff ( Neff, 2003) will be used in the current study. The test-re-test reliability score of the scale is 0.92. The scale has 12 items that are scored on a five-point Likert scale from “almost never” to “almost always”. The subscales measure self-kindness, self-judgement, humanity, isolation, and mindfulness. Average self-compassion score of 1-2.5 indicates low self-compassion, 2.5-3.5 indicates moderate, and 3.5-5.0 indicates a high level of self-compassion.

Intrinsic motivation will be measured using the Intrinsic Motivation Scale (IMS) which is developed by the investigator. The scale is developed based on Self Determination Theory ( Ryan & Deci, 2000), Mc Clelland Motivation Theory ( Pardee, 1990), and Motivation to Care Model ( Moody & Pesut, 2006). The scale’s major components are autonomy, competence, relatedness, knowledge, skills, and inner drive. There are 28 items in the IMS, which has been validated by experts with a Content Validity Index of 0.87 and a Cronbach’s alpha reliability score of 0.91.

The Perception of nursing care tool is developed by the investigator to assess the patients’ and nurse-in-charges’ perception of nursing care before and after the intervention program. The tool has ten items that are scored on a Likert scale from “strongly agree” to “strongly disagree”. The tool has been validated by experts and translated into Kannada (Local language). The Cronbach’s alpha reliability score and Content Validity Index of the tool is 0.93 and 0.87 respectively.

### 2.11 Data management

All the questionnaires are paper-and-pen based. So, the data obtained will be entered manually into Microsoft Excel (version 2306) sheets and SPSS (version 20) and will be stored under a folder with a password. The investigator will rectify the mistake if a participant signs their name or any other identifying information on the questionnaire at the time of data collection. The data will all be stored safely on a secure server for further analysis and reference, and the paper copies will be stored safely for five years after the study’s completion.

### 2.12 Statistical analysis

Mean and standard deviation will be used to describe measurement data. Frequencies and percentages will be used to represent count data. Repeated measures ANOVA will be used to analyze the effect of the training program. Chi-square will be used to see if an association between the variables before is after the training program.

### 2.13 Risk monitoring and referral

The current study is safe, considering there are no invasive procedures for the intervention. There are no direct risks associated with taking part in the current study. However, participants may feel uncomfortable or stressed while attending the program or while answering the questionnaires, of which the investigator will be very observant about. Also, in the current pandemic state, the investigator will be very vigilant and cautious of all the norms and regulations to be followed to eliminate the risk of participants getting COVID-19 infection. In any crisis, the participant will be flagged and at once referred for counselling or any other support as needed. During the study, the participants (staff nurses) are needed to attend sessions on IESE program and provide responses in the questionnaires given to them. Before recruiting the study participants, the investigator will provide them with the study’s details, including the purpose, details of the program, potential risks, and benefits of being a participant. Similarly, the patients and nurse-in-charges will also be given information in detail about the program and their role in the study.

## 3. Ethics and consent

The investigator has received approval from the university ethics committee (Kasturba Medical College and Kasturba Hospital Institutional Ethics Committee, approval number 427/2019; Institutional Ethics Committee Kasturba Medical College, Mangalore, approval number- IEC KMC MLR 08-19/331) in 2019 and has received written permission from the selected hospital authorities to conduct the study. The investigator will provide all the staff nurses, patients, and nurse-in-charges with a participant information sheet and a written consent form will be collected from the study participants. The participant information sheet will hold all the details of the study, including the aims, objectives, details about the intervention or absence of it, and the risks and benefits of participating. No one will be forced or compelled to participate in the study. The participants can withdraw from the study at any time during the study without penalty.

## 4. Dissemination

The investigator intends to disseminate the study results by publishing in peer-reviewed Scopus indexed journals and present papers at scientific conferences.

## 5. Confidentiality and access to data

The study data will be safely stored in a digital format on a secure server for five years after completion of the study. The raw data will be accessible to only the research team members and will be kept in a password-protected file. However, de-identified data can be made accessible on request.

## 6. Discussion

Nurses work in an environment that is always emotionally charged. They work with patients and their families who are most often dealing with demanding situations, emotionally and physically. Being self-aware of one’s own emotions, empathizing, having meaningful relationships, and being compassionate are important aspects of professional nursing. Understanding EI and emotion regulation skills are incredibly significant to the nursing profession because of its nature and the complexities and challenges it brings with it. Many researchers have recommended conducting studies by developing and providing a structured program to enhance nurses’ EI, IM, SC and EL. The investigator believes that the intervention program in the study would help enhance the nurses’ EI, which would influence their intrinsic motivation and self-compassion, thereby reducing their emotional labor, which would eventually positively impact their professional and personal life, as well as in the patient outcome.

## 7. Strengths and Limitations

One of the major strength of this study is that it has used established theories and models to design the conceptual framework that would guide the study. The investigator has also used established theories and models to design the intervention program which improves the credibility of the study. Another strength of this study is using multiple pre-tests and longitudinal post-test data collection method. This would be helpful in indicating the true impact of the IESE Program. However, a major limitation of the study is that there is no control arm in this quasi-experimental study. The unavailability of similar hospitals in terms of infrastructure, workforce and working schedule did not allow the investigator to select another setting that could work as a control arm. Another limitation could be a higher attrition rate due to the unpredictable nature of any hospital setting. Appropriate measures were taken to control this limitation during the sample size calculation. The investigator will also send reminder e-mails and messages to participants before every follow-up. All limitations can veil the true effect of the program. However, considering the current state of COVID-19 pandemic, where staff nurses and healthcare professionals are scarce in general, and to avoid large gatherings that can put the participants’ health at risk, the investigator believes that the current study design and sample size will suffice the study objectives.

## 8. Conclusion

The IESE program can have a significant impact on staff nurses’ personal and professional lives, which can have a positive effect on the patient care outcome. Such programs on EI and emotional well-being are a need of the hour, as healthcare workers and nurses are facing great stress in the face of COVID-19 pandemic. The findings of the current study will add to the research and development of emotional intelligence training programs among nursing and healthcare professionals.

## 9. Study status

The study intervention has been delivered to the study participants. The follow-up data collection was completed in May 2022.

## Author contributions

Saikia M conceived of the study; George LS, George A & Unnikrishnan B initiated the study design; N Ravishankar provided expertise in statistics and study design. All authors contributed to refinement of the study protocol and approved the final manuscript.

## Roles and responsibilities of committees

i. ***Principal investigator and co-authors***:
  ○ Design and conduct of the study.
  ○ Preparation of protocol and revisions
  ○ Preparation of intervention modules and materials
  ○ Publication of study reports
ii. ***Institutional Ethics Committee***:
  ○ Monitoring study
  ○ Audit of 6 monthly feedback from the principal investigator
  ○ Data verification

## Protocol amendments

Any modifications to the protocol which may impact on the conduct of the study will be informed and amended by the Institutional Ethics Committee of the current study setting.

## Data availability

### Underlying data

No underlying data are associated with this protocol.

### Extended data

figshare: Effectiveness of Integrated Emotional Self Enhancement (IESE) Program among staff nurses: protocol for a quasi-experimental study. https://doi.org/10.6084/m9.figshare.19428638.v1 ( Saikia *et al*., 2022).

The project contains the following extended data:

• English PIS (nurses).doc (Participant Information Sheet for nurses)
• English PIS (nurse-in-charge).doc (participant information sheet for nurse-in-charge)
• English PIS (patients).doc (Participant information sheet for patients)
• Socio-demographic proforma (Staff nurses).docx (demographic proforma for staff nurses)
• Demographic proforma (nurse-in-charge).docx (demographic proforma for nurse-in-charges)
• Demographic proforma (patients).docx (demographic proforma for patients)
• Intrinsic Motivation.docx (Intrinsic motivation scale, developed by the investigator)
• Nurse Incharge’s Perception.docx (nurse-in-charges’ perception of nursing care scale, developed by the investigator)
• Patients Perception.docx (patients’ perception of nursing care scale, developed by the investigator)

Data are available under the terms of the Creative Commons Attribution 4.0 International license (CC-BY 4.0).

Copyright will be applied for the booklet, workbook, and teaching material which will be developed by the investigators, and hence these materials have not been uploaded to the repository yet.

## Reporting guidelines

figshare: SPIRIT checklist for ‘Effectiveness of Integrated Emotional-Self Enhancement (IESE) Program among staff nurses: Protocol for a quasi-experimental study’. https://doi.org/10.6084/m9.figshare.19429757
