# Supplementary material for: Effectiveness of Integrated Emotional-Self Enhancement (IESE) program among staff nurses: protocol for a quasi-experimental study
Source: F1000Res. 2023 Oct 6;11:679. Originally published 2022 Jun 21. [Version 3] doi: 10.12688/f1000research.110656.3 (PMC10623541; doi:10.12688/f1000research.110656.3)
Supplement: Peer review history

# Reviewer report: Reviewer response for version 3

**DOI:** 10.5256/f1000research.154981.r212710

**Published:** 2023-11-02

**Authors:** Rafi Alnjadat

**Affiliations:** 1 Irbid University College, Al-Balqa Applied University, Irbid, Jordan

The authors have provided a nicely detailed and thorough response to the comments from the previous review and have addressed my *concerns*.

Is the study design appropriate for the research question?

Yes

Is the rationale for, and objectives of, the study clearly described?

Yes

Are sufficient details of the methods provided to allow replication by others?

Partly

Are the datasets clearly presented in a useable and accessible format?

Not applicable

Reviewer Expertise:

Clinical education, neurocognitive science

I confirm that I have read this submission and believe that I have an appropriate level of expertise to confirm that it is of an acceptable scientific standard.

# Reviewer report: Reviewer response for version 2

**DOI:** 10.5256/f1000research.136310.r189238

**Published:** 2023-07-24

**Authors:** Rafi Alnjadat

**Affiliations:** 1 Irbid University College, Al-Balqa Applied University, Irbid, Jordan

1. The introduction has a good start, but it loses its logical structure as it progresses. Please rewrite The introduction to cover all study variables.
2. Identify research gaps by reviewing existing literature.
3. Explain the importance and necessity of doing this study in India.
4. Method: Although this section is well written, it needs more detailed information.
5. Write the time of project implementation (month/year), the duration of project implementation, and the type of the hospital.
6. Add more details about the training sessions' development, the title and content of each training session, and the time of each session in a table to this section. In addition, add the content and title of educational programs provided to patients in another table and in-charge nurse if so?
7. Add more on how to control the confounding factors?
8. Explain the sample size for the patients and in-charge nurse.
9. Please highlight the limitations and strengths.
10. In the data collection section: the authors stated that "will be collected at baseline and 3-month pre-intervention, and immediately after the intervention". what is the difference between baseline and 3-months pre-intervention? kindly clarify.

Is the study design appropriate for the research question?

Yes

Is the rationale for, and objectives of, the study clearly described?

Yes

Are sufficient details of the methods provided to allow replication by others?

Partly

Are the datasets clearly presented in a useable and accessible format?

Not applicable

Reviewer Expertise:

Clinical education, neurocognitive science

I confirm that I have read this submission and believe that I have an appropriate level of expertise to confirm that it is of an acceptable scientific standard, however I have significant reservations, as outlined above.

# Response

**Published:** 2023-08-22

**Authors:** Monalisa Saikia

**Affiliations:** Manipal College of Nursing, Manipal Academy of Higher Education, Manipal, Karnataka, India

1. The investigator has modified the introduction section and have included all the study variables to make it clearer. 

 2. Research gaps were found after doing a thorough literature search. This has been added to the background section.

 3. After performing a thorough literature search, the investigator found few gaps. One of them was that the author has not found many studies that have weaved emotional well-being components of Emotional intelligence, intrinsic motivation, self-compassion and emotional labour to develop and implement a training program for nurses in India. Also, assessing perception of nursing care received by the patients and observed by the nursing in-charges/ supervisors provide a feedback and indication of the quality of care delivered by the nurses. Very few studies are available in the literature which has examined the perception of nursing care from patients and nursing supervisors after administering a training program to staff nurses. The investigator assumes that staff nurses EI, IM, SC & EL will improve after the training program which would reflect on the patients’ as well as nursing supervisors’ perception of nursing care and would eventually help in improving the staff nurses’ emotional well-being as well as patient satisfaction. As the hospital environment is stressful, especially in India where there is a shortage of nursing staff, such training programs may positively impact the staff nurses' emotional wellbeing.

 4. The investigator has added a few points to make this section more transparent.

 5. The selected hospital is a tertiary care hospital in Mangalore, India. This multispecialty hospital has outpatient and inpatient facilities that cater to patient care, teaching, and scientific research. The hospital has approximately a total of 450 staff nurses. To maintain the confidentiality of the study participants, the hospital name is not disclosed. The project was implemented from January 2021 to May 2022, with a total duration of 17 months (this period includes data collection and intervention implementation).

 6. A table (Table 2) is added to the new version which shows the outline of the training program (the content and time of each session). The investigator would like to bring this to the notice that no training program/ intervention will be provided to the patients and/or nurse-in-charges. Data about their perception of nursing care will only be collected before and after staff nurses undergo the IESE Program.

 7. The confounding factors were controlled by applying an appropriate research design, communicating clearly with the participants and stakeholders, and by making the duty roster of staff nurses well in advance so that there are lesser chances of overlap and attrition.

 8. As nurse-in-charges of all the general wards of the selected hospital will be approached for their consent to participate using complete enumeration, a sample size calculation was not performed. Similarly, a sample calculation was not performed for patients, because only those in-patients who will be receiving care from the recruited staff nurses will be approached for their consent to participate in the study. The details of the recruitment process are provided in the section 2.6-Recruitment.

 9. the strengths and limitations of this study are highlighted in Section 7 of the new version.

 10. Multiple baselines enable an assessment of whether changes occur after or during an intervention, rather than before or as a result of any extraneous factor. In the current study, the investigator assumes that at baseline, participants (staff nurses) will have little to no knowledge about the study variables. The investigator also assumes that by the time of the second pre-test, the staff nurses may gather knowledge about the study variables which may act as a bias. Thus, obtaining two pre-tests would help us identify whether there were any changes, as well as help us identify whether the changes in the post-test data are due to the intervention or due to extraneous factors.

# Reviewer report: Reviewer response for version 2

**DOI:** 10.5256/f1000research.136310.r144719

**Published:** 2022-07-27

**Authors:** Leila Bazrafkan

**Affiliations:** 1 Clinical Education Research Center, Shiraz University of Medical Sciences (SUMS), Shiraz, Iran

I think overall the paper is well written and elaborated a robust methodology.

Is the study design appropriate for the research question?

Yes

Is the rationale for, and objectives of, the study clearly described?

Partly

Are sufficient details of the methods provided to allow replication by others?

Yes

Are the datasets clearly presented in a useable and accessible format?

Not applicable

Reviewer Expertise:

medical education

I confirm that I have read this submission and believe that I have an appropriate level of expertise to confirm that it is of an acceptable scientific standard.

# Reviewer report: Reviewer response for version 1

**DOI:** 10.5256/f1000research.122283.r141764

**Published:** 2022-07-13

**Authors:** Leila Bazrafkan

**Affiliations:** 1 Clinical Education Research Center, Shiraz University of Medical Sciences (SUMS), Shiraz, Iran

The author should explain more about the intervention (integrated Emotional-Self Enhancement (IESE) program among staff nurses. How the content of the training was chosen, and whether the competence of the trainer was examined?

 Suggestion: I only suggest that since four groups are involved in implementing the intervention, they should use the study's Quasi-experimental Solomon four-group research design. I did not mean the study design is problematic, but it's merely a suggestion to improve the quality of the research design. I was considering that the author had divided the study participants into four groups. If not, the decision is up to the author, and the four-group design may have much trouble.

 Salmon's four-group design combines pre-test-post-test design with the control group and post-test design with the control group. However, when we intend to maximize the generalizability of the results of research and on the other hand, there are no clear reasons for the superiority of one of the two pre-test designs - post-test with the control group and post-test with the control group over the other. Salmon's four-group design is a logical choice.

 This method randomly divides subjects into more than three homogeneous groups. Then they will be tested. Then all of them are affected by the independent variable, the next test is taken from them, and the results are analyzed statistically. In the following figure, various comparisons of the four-group design of salmon are presented in a graphic design: *https://f1000researchdata.s3.amazonaws.com/linked/437785.solomon-four-group-comparisons.png* .

 As seen in Figure 5, groups A and B form a pre-test-post-test design with the control group, and groups C and D form a post-test design with the control group. Therefore, Solomon's four-group design is two completely independent studies analyzed in the design as mentioned above.

 In comparing the post-test of group A and the post-test of group C, i.e., comparison number 7, the effectiveness of the combination of pre-test and experimental intervention is evaluated. If the comparison of number 7 becomes meaningless, it indicates the lack of adverse effect of familiarity with the test.

 If the comparison of number 7 becomes significant, it is because the pre-test directly affected the post-test score (statistical return and familiarity with the test), or it was due to the effect of the pre-test on the experimental intervention. Suppose the difference between the post-test of groups A and B is the same as the difference between the post-test of groups C and D. In that case, there is no room to worry about the effect of the pre-test on the results, but if the differences between C5 and C1 are different, the possibility of the effect of the pre-test or interaction The pre-test (subjects) is presented with experimental intervention.

 Also, if the difference between post-test group D and B, i.e. C8 comparison, becomes meaningless, the neutrality of the pre-test effect is proven.

 However, if the mentioned comparison becomes significant, it tells about the major effect of the pre-test on the post-test. Comparing the pre-test of groups A and B with the post-test of group D is one of the most apparent aspects of salmon's four-group design. In the comparison of question C6, the difference between the pre-test of group B and the post-test of group D, becomes significant, it indicates the effect of life events and maturity.

 In fact, the effect of maturation or local history between pre-test and post-test can be measured in salmon's four-group design. For example, the occurrence of a specific social-political situation or seasonal changes may cause changes in the scores of the subjects in the post-test.

 In the comparison as mentioned above (comparison between the pre-test of groups A and B with the post-test of group D), the effect of maturity factors or life events on the post-test is measured. Maturity and life history factors are only controlled in the pre-test-post-test design with the control group, but in the Salmon design, the effect size of these factors is measured. The significance of the comparison of C3 shows the occurrence of problems in the process of random assignment.

Is the study design appropriate for the research question?

Yes

Is the rationale for, and objectives of, the study clearly described?

Partly

Are sufficient details of the methods provided to allow replication by others?

Yes

Are the datasets clearly presented in a useable and accessible format?

Not applicable

Reviewer Expertise:

medical education

I confirm that I have read this submission and believe that I have an appropriate level of expertise to confirm that it is of an acceptable scientific standard, however I have significant reservations, as outlined above.

# Response

**Published:** 2022-07-18

**Authors:** Monalisa Saikia

**Affiliations:** Manipal College of Nursing, Manipal Academy of Higher Education, Manipal, Karnataka, India

Respected Reviewer,

 Thank you for your suggestions and comments. As per your suggestion, we have added some detail about the IESE program, how it was developed, and the credibility of the investigator to deliver the program to the study participants. 

 However, your suggestion about using the Solomon Four design will not be applicable in the present study. We apologise, as we think the previous version of the manuscript failed to make this part clearer- there will be only one group of nurses, but, they will be divided into smaller groups of 5-10 members in each group so that the delivery of the training program is effective and the participants can engage in the group activities to learn more efficiently. All the nurses in each group will receive the same training program without any deviation or difference. The study investigator does not plan to make a comparison between the groups.

 Best regards.
